# 3-(4-Ferrocenylphen­yl)-1-(4-nitro­benz­yl)-1*H*-imidazol-3-ium hexa­fluorido­phosphate

**DOI:** 10.1107/S2414314626002713

**Published:** 2026-03-27

**Authors:** Halliru Ibrahim, Sizwe J. Zamisa, Muhammad D. Bala, Pinkie Ntola

**Affiliations:** a Department of Chemistry, Durban University of Technology, PO Box 1334, Durban, 4000, South Africa; b Discipline of Chemistry, University of KwaZulu-Natal, Private Bag X54001, Westville, Durban, 4000, South Africa

**Keywords:** crystal structure, imidazolium salt, ferrocenylphen­yl

## Abstract

The asymmetric unit of the title salt, [Fe(C_5_H_5_)(C_21_H_17_N_3_O_2_]PF_6_, comprises a 1-(4-ferrocenylphen­yl)-3-(4-nitro­benz­yl)imidazolium cation paired with a hexa­fluorido­phosphate anion. The cation adopts a *syn*-periplanar arrangement, with the ferrocenylphenyl substituent tilted by 38.32 (7)° relative to the imidazolium core. The ferrocenyl fragment shows a nearly eclipsed conformation defined by a C(Cp)—*Cg*(Cp1)—*Cg*(Cp2)—C(Cp) torsion angle of −7.9° where *Cg*1 and *Cg*2 are the centroids of the cyclo­penta­dienyl rings of the ferrocenyl substituent. In the crystal, inter­ionic C—H⋯F hydrogen bonds generate graph-set motifs *R*^1^_2_(7) and *R*^2^_1_(4), assembling the ions into chains that propagate parallel to [001].

## Abstract 2

In the title ferrocenylphenyl functionalized imidazolium salt, the 1-(4-ferrocenylphen­yl)-3-(4-nitro­benz­yl)imidazolium cation adopts a *syn*-periplanar arrangement, with the ferrocenylphenyl substituent tilted by 38.32 (7)° relative to the imidazolium core. In the crystal, inter­ionic C—H⋯F hydrogen bonds generate graph-set motifs *R*^1^_2_(7) and *R*^2^_1_(4), assembling the ions into chains that propagate along the *c-*axis direction.

## Graphical abstract

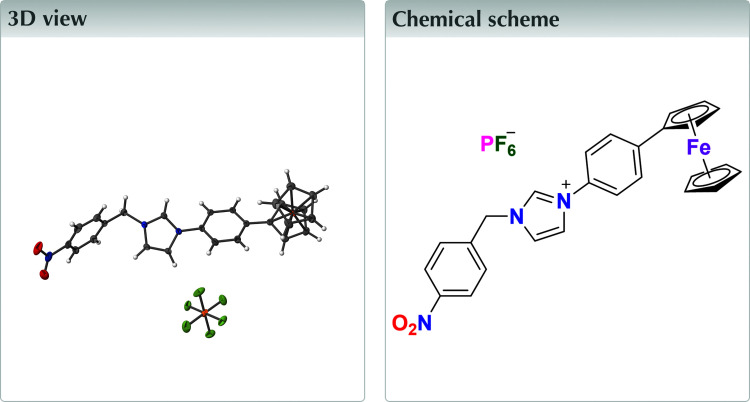

## Structure description

The title compound is a new ferrocenylphenyl functionalized imidazolium salt, synthesized by the quaternization of 1-(4-ferrocen­ylphen­yl)imidazole with *p*-nitro­benzyl­bromide. The mol­ecular structures of analogous species to the title compound are relatively rare (Onyancha *et al.*, 2010 ▸), with related analogues having a methyl­ene spacer between the ferrocenyl and imidazolyl moieties (Ikhile *et al.*, 2013 ▸; Ndlovu *et al.*, 2017 ▸). Modifications in the *N*-substituents on the imidazolyl moieties with groups containing ferrocenyl and associated 4,5-aryl substituents in their design have been studied for their unique steric and electronic properties (Diaz de Greñu *et al.*, 2023 ▸; Krishnanjaneyulu *et al.*, 2014 ▸). There are reviews available covering the biological activity of the heteroatom-functionalized (Ibrahim *et al.*, 2025 ▸) and non-heteroatom functionalized azolium salts (Patil *et al.*, 2020 ▸; Fletcher *et al.*, 2018 ▸; Mercs & Albrecht, 2010 ▸), which have provided evidence on the structure–activity trends in their well-established potential as anti-fungal, anti-bacterial and anti-proliferative agents. Furthermore, the stability and non-toxicity of the ferrocenium salts (Fouda *et al.*, 2007 ▸; Patra & Gasser, 2017 ▸) have contributed to the growing inter­est in the development of new and more biologically active ferrocenylimidazolium salts (Larik *et al.*, 2017 ▸; Zampino *et al.*, 2021 ▸). As part of our work in developing new imidazolium derivatives with anti-microbial activities (Kadafour *et al.*, 2022 ▸; Ndlovu *et al.*, 2017 ▸), we synthesized the title compound and analysed its crystal structure.

**Figure 1:**
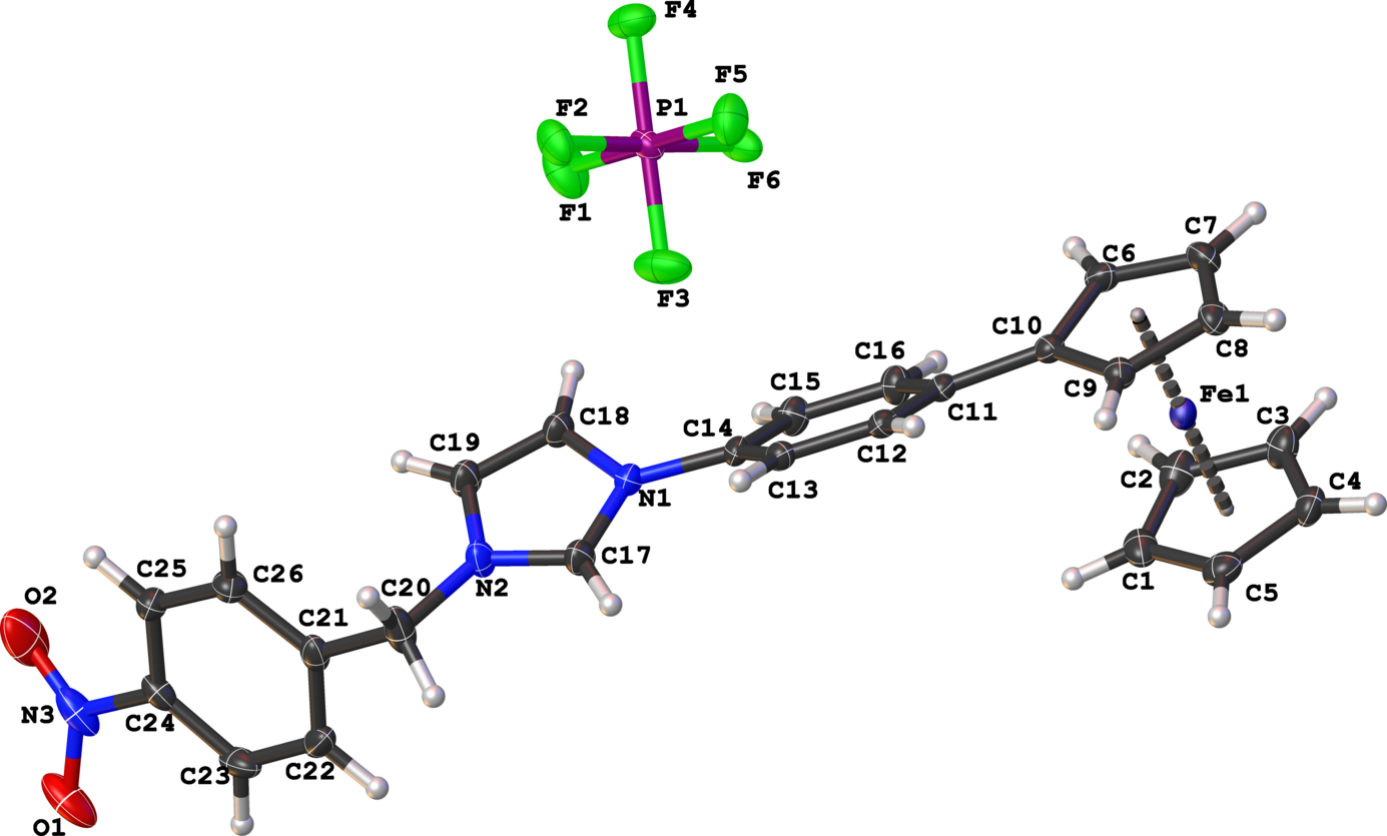
The mol­ecular structure of the title salt showing the atom-numbering scheme and displacement ellipsoids drawn at the 50% probability level.

**Figure 2:**
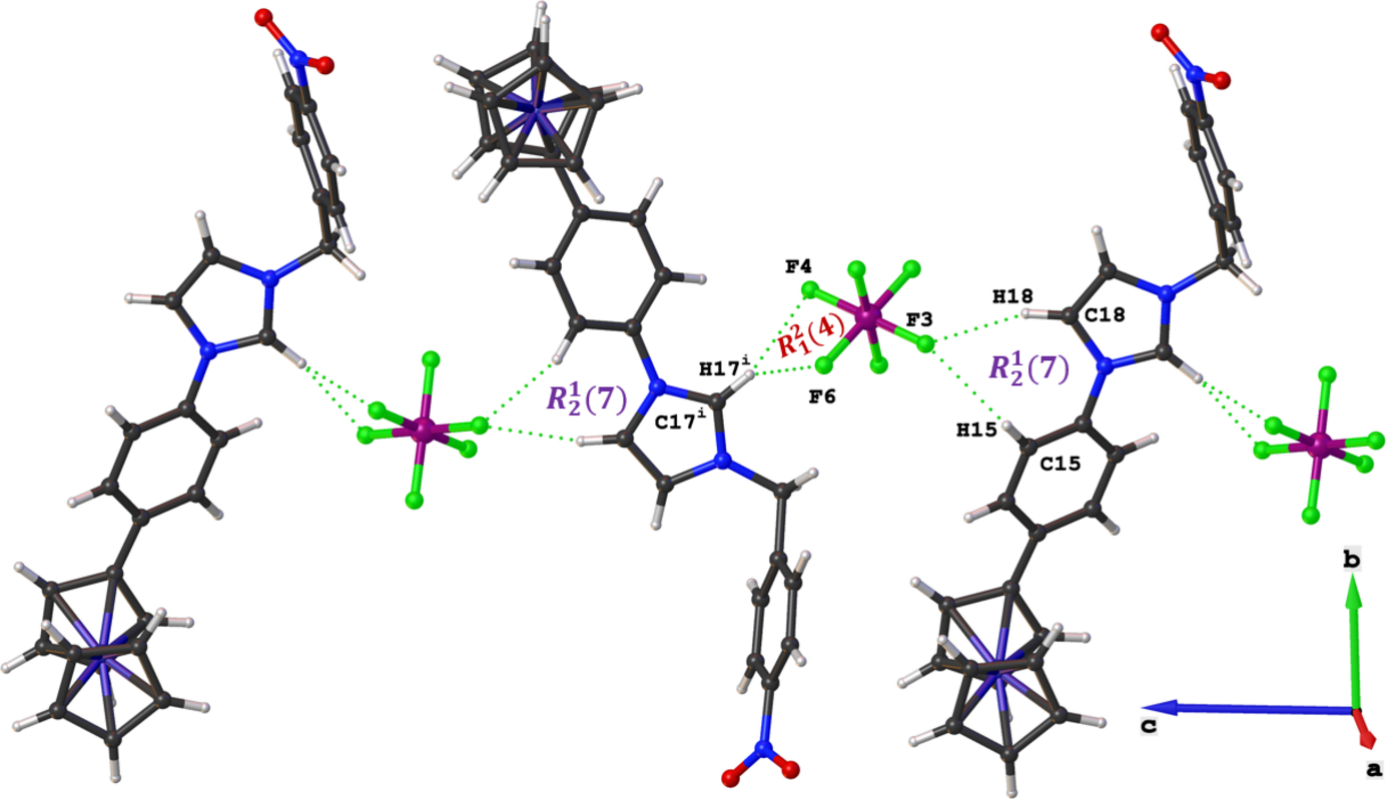
A representation of the C—H⋯F hydrogen bonds in the crystal of the title compound.

The asymmetric unit of the title compound has a cationic 1-(4-ferrocenylphen­yl)-3-(4-nitro­benz­yl)imidazolium species and a PF_6_^−^ counter-ion (Fig. 1 ▸). The imidazolium cation adopts a *syn*-periplanar conformation, with the 4-ferrocenylphenyl rings inclined to the central imidazolium ring by 38.32 (7)°. The ferrocenyl moiety exhibits a near eclipsed conformation with a C1—*Cg*(Cp ring1)—*Cg*(Cp ring2)—C10 angle of −7.9°, which is similar to related ferrocenylphenyl imidazolium salts (Mochida *et al.*, 2011 ▸; Horváth *et al.*, 2008 ▸; Onyancha *et al.*, 2010 ▸); Cp = cyclo­penta­dienyl. Inter­molecular C—H⋯F hydrogen-bonding patterns, with graph-set descriptors 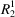
(7) and 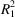
(4), exist between neighbouring ionic species to form a supra­molecular chain parallel to [001] (Table 1 ▸ and Fig. 2 ▸).

## Synthesis and crystallization

The synthesis of the title compound was carried out by an adaptation of the protocol used for its analogues bearing *N-*substituted *p*-NO_2_-phenyl moiety (Ibrahim *et al.*, 2024 ▸). To a Schlenck tube initially charged with 1-(4-ferrocenylphen­yl)imidazole and an excess of *p*-nitro­benzyl bromide (1.3 mole equivalent) was added dry aceto­nitrile (20 ml). The mixture was stirred and refluxed under nitro­gen for 16 h. The removal of all volatiles from the dark-brown solution gave a crude brown product which after elution with a gradient of solvent mixtures (diethyl ether, DCM and ethyl acetate) in a column gave the bromide salt as eluent of DCM/ethyl acetate (3:2), which after vacuum removal of the solvent gave a pink–orange microcrystalline powder. Anionic metathesis with KPF_6_ (1 mole equivalent) in methanol and the subsequent workup afforded the title compound as a yellow–orange, air-stable microcrystalline powder. The hexa­fluorido­phosphate salt is insoluble in dry methanol and dry DCM, but dissolves upon the addition of a few drops of DCM to its suspension in methanol. Yield: 0.24 g, 0.4 mmol, 65.3%. M.p. 158–160 °C. ^1^H NMR (400 MHz, DMSO-*d*_6_): δ 10.03 (*s*, 1H, NCHN), 8.39 [*s*, 1H, C*H*=C(imid)], 8.30 [*d*, *J* = 8.7 Hz, 2H, 2 *x* 1H, CH(benz­yl)], 8.06 [*s*, 1H, C=C*H*(imid)], 7.79 [*m*, 4H, 4 × 1H, CH(phen­yl)], 7.69 [*m*, 2H, 2 × 1H, CH(benz­yl)], 5.69 (*s*, 2H, CH_2_—N), 4.94 [*d*, *J* = 3.3 Hz, 2H, 2 × 1H, CH(Cp)], 4.44 [*d*, *J* = 1.4 Hz, 2H, 2 × 1H, CH(Cp)], 4.04 [*s*, 5H, 5 × 1H, CH(Cp)]. ^31^P (400 MHz, DMSO-d_6_): δ 135–152 (m, PF_6_^−^). Crystals suitable for the X-ray diffraction study were grown by the slow diffusion of hexane into a MeOH/DCM solution of the title compound.

## Refinement

Crystal data, data collection and structure refinement details are summarized in Table 2 ▸.

## Supplementary Material

Crystal structure: contains datablock(s) I. DOI: 10.1107/S2414314626002713/tk4123sup1.cif

Structure factors: contains datablock(s) I. DOI: 10.1107/S2414314626002713/tk4123Isup2.hkl

CCDC reference: 2537159

Additional supporting information:  crystallographic information; 3D view; checkCIF report

## Figures and Tables

**Table 1:** Hydrogen-bond geometry (Å, °)

*D*—H⋯*A*	*D*—H	H⋯*A*	*D*⋯*A*	*D*—H⋯*A*
C17—H17⋯F4^i^	0.95	2.29	3.208 (2)	163
C17—H17⋯F6^i^	0.95	2.34	3.115 (2)	138
C18—H18⋯F3	0.95	2.30	3.207 (2)	158
C15—H15⋯F3	0.95	2.53	3.216 (2)	129

Symmetry code: (i) 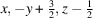.

**Table 2:** Experimental details

Crystal data	
Chemical formula	[Fe(C_5_H_5_)(C_21_H_17_N_3_O_2_)]PF_6_
*M*_r_	609.28
Crystal system, space group	Monoclinic, *P*2_1_/*c*
Temperature (K)	100
*a*, *b*, *c* (Å)	10.6952 (3), 11.9279 (3), 19.7519 (5)
β (°)	104.450 (1)
*V* (Å^3^)	2440.06 (11)
*Z*	4
Radiation type	Mo *K*α
μ (mm^−1^)	0.76
Crystal size (mm)	0.34 × 0.29 × 0.17

Data collection	
Diffractometer	Bruker APEXII CCD
Absorption correction	Multi-scan (*SADABS*; Krause *et al.*, 2015 ▸)
*T*_min_, *T*_max_	0.674, 0.746
No. of measured, independent and observed [*I* > 2σ(*I*)] reflections	19608, 5986, 5068
*R*_int_	0.026
(sin θ/λ)_max_ (Å^−1^)	0.666

Refinement	
*R*[*F*^2^ > 2σ(*F*^2^)], *wR*(*F*^2^), *S*	0.035, 0.087, 1.04
No. of reflections	5986
No. of parameters	352
H-atom treatment	H-atom parameters constrained
Δρ_max_, Δρ_min_ (e Å^−3^)	0.56, −0.34

Computer programs: *APEX2* and *SAINT* (Bruker, 2009 ▸), *SHELXS2013* (Sheldrick, 2008 ▸), *SHELXL2018/3* (Sheldrick, 2015 ▸) and *OLEX2* (Dolomanov *et al.*, 2009 ▸).

## full crystallographic data

### 3-(4-Ferrocenylphenyl)-1-(4-nitrobenzyl)-1H-imidazol-3-ium hexafluoridophosphate Crystal data

**Table:**

[Fe(C_5_H_5_)(C_21_H_17_N_3_O_2_)]PF_6_	*F*(000) = 1240
*M**_r_* = 609.28	*D*_x_ = 1.659 Mg m^−^^3^
Monoclinic, *P*2_1_/*c*	Mo *K*α radiation, λ = 0.71073 Å
*a* = 10.6952 (3) Å	Cell parameters from 7894 reflections
*b* = 11.9279 (3) Å	θ = 2.6–28.5°
*c* = 19.7519 (5) Å	µ = 0.76 mm^−^^1^
β = 104.450 (1)°	*T* = 100 K
*V* = 2440.06 (11) Å^3^	Block, red
*Z* = 4	0.34 × 0.29 × 0.17 mm

### 3-(4-Ferrocenylphenyl)-1-(4-nitrobenzyl)-1H-imidazol-3-ium hexafluoridophosphate Data collection

**Table:**

Bruker APEXII CCD diffractometer	5986 independent reflections
Radiation source: microfocus sealed X-ray tube, Incoatec Iµs	5068 reflections with *I* > 2σ(*I*)
Mirror optics monochromator	*R*_int_ = 0.026
Detector resolution: 7.9 pixels mm^-1^	θ_max_ = 28.3°, θ_min_ = 2.0°
ω and φ scans	*h* = −14→14
Absorption correction: multi-scan (SADABS; Krause *et al.*, 2015)	*k* = −15→15
*T*_min_ = 0.674, *T*_max_ = 0.746	*l* = −13→26
19608 measured reflections	

### 3-(4-Ferrocenylphenyl)-1-(4-nitrobenzyl)-1H-imidazol-3-ium hexafluoridophosphate Refinement

**Table:**

Refinement on *F*^2^	0 restraints
Least-squares matrix: full	Hydrogen site location: inferred from neighbouring sites
*R*[*F*^2^ > 2σ(*F*^2^)] = 0.035	H-atom parameters constrained
*wR*(*F*^2^) = 0.087	*w* = 1/[σ^2^(*F*_o_^2^) + (0.0381*P*)^2^ + 1.6154*P*] where *P* = (*F*_o_^2^ + 2*F*_c_^2^)/3
*S* = 1.04	(Δ/σ)_max_ = 0.002
5986 reflections	Δρ_max_ = 0.56 e Å^−^^3^
352 parameters	Δρ_min_ = −0.34 e Å^−^^3^

### 3-(4-Ferrocenylphenyl)-1-(4-nitrobenzyl)-1H-imidazol-3-ium hexafluoridophosphate Special details

**Table:**

Geometry. All esds (except the esd in the dihedral angle between two l.s. planes) are estimated using the full covariance matrix. The cell esds are taken into account individually in the estimation of esds in distances, angles and torsion angles; correlations between esds in cell parameters are only used when they are defined by crystal symmetry. An approximate (isotropic) treatment of cell esds is used for estimating esds involving l.s. planes.

### 3-(4-Ferrocenylphenyl)-1-(4-nitrobenzyl)-1H-imidazol-3-ium hexafluoridophosphate Fractional atomic coordinates and isotropic or equivalent isotropic displacement parameters (Å^2^)

**Table:**

	*x*	*y*	*z*	*U*_iso_*/*U*_eq_	
Fe1	0.30221 (2)	0.22080 (2)	0.55995 (2)	0.01655 (7)	
P1	0.19702 (4)	0.86596 (4)	0.68883 (2)	0.01988 (11)	
F1	0.27912 (13)	0.97795 (11)	0.70552 (7)	0.0430 (3)	
F2	0.08662 (11)	0.92940 (10)	0.63247 (6)	0.0306 (3)	
F3	0.26959 (12)	0.83520 (13)	0.63015 (6)	0.0411 (3)	
F4	0.12400 (12)	0.89624 (11)	0.74852 (6)	0.0342 (3)	
F5	0.11528 (12)	0.75246 (10)	0.67333 (7)	0.0353 (3)	
F6	0.30462 (11)	0.80055 (11)	0.74615 (6)	0.0313 (3)	
C12	0.09883 (16)	0.47972 (14)	0.43813 (9)	0.0174 (3)	
H12	0.043262	0.425396	0.410917	0.021*	
C13	0.10591 (17)	0.58648 (14)	0.41122 (9)	0.0183 (3)	
H13	0.055956	0.605277	0.365761	0.022*	
O1	0.52346 (15)	1.41649 (15)	0.30758 (9)	0.0448 (4)	
C14	0.18672 (16)	0.66518 (14)	0.45152 (9)	0.0181 (3)	
N1	0.19046 (14)	0.77750 (12)	0.42541 (8)	0.0185 (3)	
N2	0.18694 (16)	0.91627 (12)	0.35471 (8)	0.0216 (3)	
C20	0.1721 (2)	0.97812 (16)	0.28811 (10)	0.0309 (5)	
H20A	0.079119	0.990629	0.266677	0.037*	
H20B	0.207036	0.932009	0.255402	0.037*	
C9	0.11715 (16)	0.23877 (14)	0.49861 (10)	0.0182 (3)	
H9	0.089577	0.230299	0.449294	0.022*	
C10	0.16144 (16)	0.34054 (14)	0.53527 (9)	0.0172 (3)	
C6	0.19259 (17)	0.31526 (15)	0.60828 (9)	0.0208 (4)	
H6	0.224686	0.366992	0.645089	0.025*	
C7	0.16754 (19)	0.19999 (16)	0.61658 (10)	0.0244 (4)	
H7	0.179336	0.161241	0.659734	0.029*	
C8	0.12167 (17)	0.15272 (15)	0.54888 (10)	0.0222 (4)	
H8	0.098051	0.076578	0.538938	0.027*	
C11	0.17252 (16)	0.45147 (14)	0.50472 (9)	0.0169 (3)	
C17	0.18432 (19)	0.80510 (15)	0.35957 (10)	0.0222 (4)	
H17	0.178932	0.753924	0.322099	0.027*	
C19	0.19581 (18)	0.96098 (15)	0.42007 (10)	0.0224 (4)	
H19	0.199428	1.038372	0.431765	0.027*	
C18	0.19839 (18)	0.87456 (15)	0.46431 (10)	0.0213 (4)	
H18	0.204499	0.879477	0.513034	0.026*	
C21	0.24078 (19)	1.08959 (15)	0.29854 (9)	0.0222 (4)	
C26	0.17793 (18)	1.18339 (16)	0.31577 (10)	0.0226 (4)	
H26	0.091682	1.176627	0.319971	0.027*	
C25	0.23894 (18)	1.28616 (15)	0.32688 (10)	0.0226 (4)	
H25	0.197014	1.349735	0.340081	0.027*	
C24	0.36235 (18)	1.29360 (15)	0.31824 (10)	0.0221 (4)	
N3	0.42953 (18)	1.40204 (16)	0.33161 (9)	0.0329 (4)	
O2	0.3897 (2)	1.47048 (14)	0.36698 (10)	0.0500 (5)	
C23	0.42634 (19)	1.20348 (18)	0.29915 (11)	0.0283 (4)	
H23	0.510674	1.211822	0.292302	0.034*	
C22	0.3650 (2)	1.10048 (17)	0.29015 (10)	0.0272 (4)	
H22	0.408295	1.036806	0.278151	0.033*	
C15	0.26122 (18)	0.63900 (15)	0.51743 (10)	0.0220 (4)	
H15	0.316544	0.693634	0.544475	0.026*	
C16	0.25419 (17)	0.53241 (15)	0.54347 (10)	0.0216 (4)	
H16	0.305880	0.513838	0.588567	0.026*	
C2	0.48705 (18)	0.27324 (16)	0.60214 (11)	0.0276 (4)	
H2	0.512690	0.333833	0.633779	0.033*	
C1	0.45757 (18)	0.28022 (16)	0.52837 (11)	0.0271 (4)	
H1	0.459275	0.346409	0.501868	0.033*	
C5	0.42492 (18)	0.17093 (17)	0.50072 (11)	0.0258 (4)	
H5	0.401584	0.151137	0.452617	0.031*	
C4	0.43347 (18)	0.09662 (15)	0.55800 (11)	0.0247 (4)	
H4	0.416593	0.018350	0.554874	0.030*	
C3	0.47169 (18)	0.15990 (16)	0.62076 (11)	0.0261 (4)	
H3	0.484649	0.131493	0.666924	0.031*	

### 3-(4-Ferrocenylphenyl)-1-(4-nitrobenzyl)-1H-imidazol-3-ium hexafluoridophosphate Atomic displacement parameters (Å^2^)

**Table:**

	*U*^11^	*U*^22^	*U*^33^	*U*^12^	*U*^13^	*U*^23^
Fe1	0.01709 (13)	0.01395 (12)	0.01845 (13)	0.00149 (9)	0.00414 (9)	0.00176 (9)
P1	0.0170 (2)	0.0248 (2)	0.0177 (2)	0.00035 (17)	0.00410 (17)	0.00656 (18)
F1	0.0370 (7)	0.0374 (7)	0.0469 (8)	−0.0159 (6)	−0.0037 (6)	0.0075 (6)
F2	0.0257 (6)	0.0328 (6)	0.0295 (6)	0.0016 (5)	−0.0001 (5)	0.0135 (5)
F3	0.0331 (7)	0.0700 (9)	0.0226 (6)	0.0097 (7)	0.0114 (5)	0.0068 (6)
F4	0.0316 (6)	0.0475 (7)	0.0257 (6)	0.0117 (6)	0.0111 (5)	0.0020 (5)
F5	0.0325 (7)	0.0232 (6)	0.0469 (8)	−0.0010 (5)	0.0036 (6)	0.0028 (5)
F6	0.0215 (6)	0.0481 (7)	0.0247 (6)	0.0091 (5)	0.0063 (5)	0.0153 (5)
C12	0.0178 (8)	0.0164 (8)	0.0191 (9)	0.0018 (6)	0.0071 (7)	−0.0011 (6)
C13	0.0198 (8)	0.0188 (8)	0.0172 (8)	0.0036 (7)	0.0064 (7)	0.0009 (6)
O1	0.0329 (8)	0.0591 (11)	0.0374 (9)	−0.0228 (8)	−0.0006 (7)	0.0210 (8)
C14	0.0200 (8)	0.0128 (8)	0.0236 (9)	0.0039 (6)	0.0095 (7)	0.0020 (6)
N1	0.0211 (7)	0.0144 (7)	0.0210 (8)	0.0019 (6)	0.0071 (6)	0.0000 (6)
N2	0.0304 (8)	0.0149 (7)	0.0195 (8)	−0.0013 (6)	0.0060 (6)	0.0008 (6)
C20	0.0532 (13)	0.0182 (9)	0.0190 (10)	−0.0030 (9)	0.0049 (9)	0.0019 (7)
C9	0.0162 (8)	0.0178 (8)	0.0208 (9)	−0.0001 (6)	0.0048 (7)	0.0012 (7)
C10	0.0157 (8)	0.0181 (8)	0.0187 (8)	0.0026 (6)	0.0058 (6)	0.0010 (6)
C6	0.0231 (9)	0.0227 (9)	0.0182 (9)	0.0035 (7)	0.0082 (7)	−0.0008 (7)
C7	0.0272 (10)	0.0256 (9)	0.0231 (9)	0.0030 (8)	0.0114 (8)	0.0076 (7)
C8	0.0200 (9)	0.0191 (8)	0.0290 (10)	−0.0014 (7)	0.0085 (7)	0.0035 (7)
C11	0.0161 (8)	0.0157 (8)	0.0203 (9)	0.0028 (6)	0.0075 (6)	0.0008 (6)
C17	0.0295 (10)	0.0155 (8)	0.0228 (9)	0.0000 (7)	0.0086 (7)	−0.0010 (7)
C19	0.0293 (10)	0.0159 (8)	0.0220 (9)	−0.0001 (7)	0.0061 (7)	−0.0024 (7)
C18	0.0269 (9)	0.0162 (8)	0.0208 (9)	0.0007 (7)	0.0061 (7)	−0.0026 (7)
C21	0.0326 (10)	0.0169 (8)	0.0170 (9)	0.0015 (7)	0.0059 (7)	0.0033 (7)
C26	0.0194 (8)	0.0226 (9)	0.0273 (10)	−0.0006 (7)	0.0087 (7)	0.0022 (7)
C25	0.0247 (9)	0.0179 (8)	0.0276 (10)	0.0030 (7)	0.0109 (7)	0.0017 (7)
C24	0.0228 (9)	0.0234 (9)	0.0196 (9)	−0.0052 (7)	0.0045 (7)	0.0062 (7)
N3	0.0354 (10)	0.0343 (10)	0.0249 (9)	−0.0153 (8)	−0.0003 (7)	0.0100 (8)
O2	0.0765 (13)	0.0297 (9)	0.0444 (10)	−0.0215 (9)	0.0163 (9)	−0.0058 (8)
C23	0.0201 (9)	0.0403 (12)	0.0274 (10)	0.0053 (8)	0.0115 (8)	0.0121 (8)
C22	0.0361 (11)	0.0262 (10)	0.0232 (10)	0.0144 (8)	0.0147 (8)	0.0065 (8)
C15	0.0215 (9)	0.0167 (8)	0.0261 (10)	−0.0010 (7)	0.0026 (7)	−0.0022 (7)
C16	0.0219 (9)	0.0192 (8)	0.0212 (9)	0.0019 (7)	0.0006 (7)	0.0023 (7)
C2	0.0177 (9)	0.0221 (9)	0.0387 (12)	0.0006 (7)	−0.0010 (8)	−0.0034 (8)
C1	0.0179 (9)	0.0249 (9)	0.0406 (12)	0.0021 (7)	0.0109 (8)	0.0082 (8)
C5	0.0209 (9)	0.0306 (10)	0.0266 (10)	0.0064 (8)	0.0073 (7)	−0.0006 (8)
C4	0.0224 (9)	0.0174 (9)	0.0340 (11)	0.0049 (7)	0.0064 (8)	0.0006 (8)
C3	0.0240 (9)	0.0239 (9)	0.0270 (10)	0.0068 (7)	0.0000 (8)	0.0029 (8)

### 3-(4-Ferrocenylphenyl)-1-(4-nitrobenzyl)-1H-imidazol-3-ium hexafluoridophosphate Geometric parameters (Å, º)

**Table:**

Fe1—C9	2.0578 (17)	C10—C11	1.471 (2)
Fe1—C10	2.0440 (17)	C6—H6	0.9500
Fe1—C6	2.0284 (18)	C6—C7	1.418 (3)
Fe1—C7	2.0473 (19)	C7—H7	0.9500
Fe1—C8	2.0555 (18)	C7—C8	1.421 (3)
Fe1—C2	2.0425 (19)	C8—H8	0.9500
Fe1—C1	2.0419 (19)	C11—C16	1.395 (2)
Fe1—C5	2.0520 (19)	C17—H17	0.9500
Fe1—C4	2.0477 (18)	C19—H19	0.9500
Fe1—C3	2.0419 (18)	C19—C18	1.347 (3)
P1—F1	1.5873 (13)	C18—H18	0.9500
P1—F2	1.5969 (11)	C21—C26	1.390 (3)
P1—F3	1.5905 (13)	C21—C22	1.385 (3)
P1—F4	1.6092 (13)	C26—H26	0.9500
P1—F5	1.5992 (13)	C26—C25	1.380 (3)
P1—F6	1.6015 (11)	C25—H25	0.9500
C12—H12	0.9500	C25—C24	1.375 (3)
C12—C13	1.389 (2)	C24—N3	1.471 (2)
C12—C11	1.396 (2)	C24—C23	1.377 (3)
C13—H13	0.9500	N3—O2	1.218 (3)
C13—C14	1.385 (2)	C23—H23	0.9500
O1—N3	1.226 (2)	C23—C22	1.383 (3)
C14—N1	1.440 (2)	C22—H22	0.9500
C14—C15	1.382 (3)	C15—H15	0.9500
N1—C17	1.327 (2)	C15—C16	1.380 (2)
N1—C18	1.380 (2)	C16—H16	0.9500
N2—C20	1.482 (2)	C2—H2	0.9500
N2—C17	1.330 (2)	C2—C1	1.414 (3)
N2—C19	1.378 (2)	C2—C3	1.421 (3)
C20—H20A	0.9900	C1—H1	0.9500
C20—H20B	0.9900	C1—C5	1.423 (3)
C20—C21	1.508 (3)	C5—H5	0.9500
C9—H9	0.9500	C5—C4	1.422 (3)
C9—C10	1.432 (2)	C4—H4	0.9500
C9—C8	1.421 (2)	C4—C3	1.421 (3)
C10—C6	1.429 (2)	C3—H3	0.9500
			
C10—Fe1—C9	40.87 (7)	C6—C10—C9	107.21 (15)
C10—Fe1—C7	68.79 (7)	C6—C10—C11	125.54 (16)
C10—Fe1—C8	68.54 (7)	C11—C10—Fe1	126.61 (12)
C10—Fe1—C5	127.19 (7)	Fe1—C6—H6	125.5
C10—Fe1—C4	165.60 (8)	C10—C6—Fe1	70.04 (10)
C6—Fe1—C9	68.61 (7)	C10—C6—H6	125.7
C6—Fe1—C10	41.08 (7)	C7—C6—Fe1	70.35 (10)
C6—Fe1—C7	40.72 (7)	C7—C6—C10	108.54 (16)
C6—Fe1—C8	68.38 (8)	C7—C6—H6	125.7
C6—Fe1—C2	104.60 (8)	Fe1—C7—H7	126.5
C6—Fe1—C1	124.12 (8)	C6—C7—Fe1	68.93 (10)
C6—Fe1—C5	163.01 (8)	C6—C7—H7	126.1
C6—Fe1—C4	152.89 (8)	C6—C7—C8	107.87 (16)
C6—Fe1—C3	116.95 (8)	C8—C7—Fe1	70.05 (11)
C7—Fe1—C9	68.28 (8)	C8—C7—H7	126.1
C7—Fe1—C8	40.53 (8)	Fe1—C8—H8	126.4
C7—Fe1—C5	156.00 (8)	C9—C8—Fe1	69.88 (10)
C7—Fe1—C4	120.11 (8)	C9—C8—C7	108.34 (16)
C8—Fe1—C9	40.41 (7)	C9—C8—H8	125.8
C2—Fe1—C9	154.04 (8)	C7—C8—Fe1	69.43 (11)
C2—Fe1—C10	117.62 (7)	C7—C8—H8	125.8
C2—Fe1—C7	123.74 (9)	C12—C11—C10	121.29 (16)
C2—Fe1—C8	162.23 (8)	C16—C11—C12	118.61 (16)
C2—Fe1—C5	68.27 (8)	C16—C11—C10	120.05 (16)
C2—Fe1—C4	68.34 (8)	N1—C17—N2	108.74 (16)
C1—Fe1—C9	121.36 (8)	N1—C17—H17	125.6
C1—Fe1—C10	106.96 (7)	N2—C17—H17	125.6
C1—Fe1—C7	161.09 (8)	N2—C19—H19	126.4
C1—Fe1—C8	156.79 (8)	C18—C19—N2	107.28 (16)
C1—Fe1—C2	40.52 (9)	C18—C19—H19	126.4
C1—Fe1—C5	40.67 (8)	N1—C18—H18	126.5
C1—Fe1—C4	68.37 (8)	C19—C18—N1	106.99 (16)
C5—Fe1—C9	110.64 (8)	C19—C18—H18	126.5
C5—Fe1—C8	122.84 (8)	C26—C21—C20	119.61 (18)
C4—Fe1—C9	128.87 (8)	C22—C21—C20	121.02 (18)
C4—Fe1—C8	110.03 (8)	C22—C21—C26	119.37 (17)
C4—Fe1—C5	40.59 (8)	C21—C26—H26	119.5
C3—Fe1—C9	164.94 (7)	C25—C26—C21	121.02 (17)
C3—Fe1—C10	151.85 (8)	C25—C26—H26	119.5
C3—Fe1—C7	106.13 (8)	C26—C25—H25	121.1
C3—Fe1—C8	126.48 (8)	C24—C25—C26	117.90 (17)
C3—Fe1—C2	40.73 (8)	C24—C25—H25	121.1
C3—Fe1—C1	68.44 (8)	C25—C24—N3	118.39 (18)
C3—Fe1—C5	68.41 (8)	C25—C24—C23	122.80 (18)
C3—Fe1—C4	40.68 (8)	C23—C24—N3	118.80 (18)
F1—P1—F2	91.17 (7)	O1—N3—C24	117.71 (19)
F1—P1—F3	90.01 (8)	O2—N3—O1	124.42 (19)
F1—P1—F4	90.20 (8)	O2—N3—C24	117.85 (18)
F1—P1—F5	179.05 (8)	C24—C23—H23	120.8
F1—P1—F6	90.03 (7)	C24—C23—C22	118.45 (18)
F2—P1—F4	89.70 (7)	C22—C23—H23	120.8
F2—P1—F5	89.65 (7)	C21—C22—H22	119.8
F2—P1—F6	178.37 (7)	C23—C22—C21	120.42 (18)
F3—P1—F2	90.67 (7)	C23—C22—H22	119.8
F3—P1—F4	179.57 (8)	C14—C15—H15	120.4
F3—P1—F5	90.46 (8)	C16—C15—C14	119.14 (17)
F3—P1—F6	90.43 (7)	C16—C15—H15	120.4
F5—P1—F4	89.32 (7)	C11—C16—H16	119.4
F5—P1—F6	89.14 (7)	C15—C16—C11	121.18 (17)
F6—P1—F4	89.19 (6)	C15—C16—H16	119.4
C13—C12—H12	119.7	Fe1—C2—H2	126.3
C13—C12—C11	120.67 (16)	C1—C2—Fe1	69.72 (11)
C11—C12—H12	119.7	C1—C2—H2	125.9
C12—C13—H13	120.4	C1—C2—C3	108.18 (17)
C14—C13—C12	119.15 (16)	C3—C2—Fe1	69.61 (11)
C14—C13—H13	120.4	C3—C2—H2	125.9
C13—C14—N1	119.60 (16)	Fe1—C1—H1	125.9
C15—C14—C13	121.24 (16)	C2—C1—Fe1	69.76 (11)
C15—C14—N1	119.13 (16)	C2—C1—H1	125.9
C17—N1—C14	125.65 (15)	C2—C1—C5	108.17 (17)
C17—N1—C18	108.58 (15)	C5—C1—Fe1	70.04 (11)
C18—N1—C14	125.76 (15)	C5—C1—H1	125.9
C17—N2—C20	124.11 (16)	Fe1—C5—H5	126.6
C17—N2—C19	108.42 (15)	C1—C5—Fe1	69.28 (11)
C19—N2—C20	127.31 (15)	C1—C5—H5	126.1
N2—C20—H20A	109.2	C4—C5—Fe1	69.54 (11)
N2—C20—H20B	109.2	C4—C5—C1	107.74 (18)
N2—C20—C21	112.15 (16)	C4—C5—H5	126.1
H20A—C20—H20B	107.9	Fe1—C4—H4	126.3
C21—C20—H20A	109.2	C5—C4—Fe1	69.87 (10)
C21—C20—H20B	109.2	C5—C4—H4	126.0
Fe1—C9—H9	126.8	C3—C4—Fe1	69.44 (11)
C10—C9—Fe1	69.05 (10)	C3—C4—C5	108.08 (17)
C10—C9—H9	126.0	C3—C4—H4	126.0
C8—C9—Fe1	69.71 (10)	Fe1—C3—H3	125.9
C8—C9—H9	126.0	C2—C3—Fe1	69.66 (11)
C8—C9—C10	108.04 (16)	C2—C3—H3	126.1
C9—C10—Fe1	70.08 (10)	C4—C3—Fe1	69.88 (11)
C9—C10—C11	127.24 (16)	C4—C3—C2	107.83 (18)
C6—C10—Fe1	68.87 (10)	C4—C3—H3	126.1
			
Fe1—C9—C10—C6	−59.10 (12)	C6—C10—C11—C12	−155.93 (17)
Fe1—C9—C10—C11	121.42 (17)	C6—C10—C11—C16	21.6 (3)
Fe1—C9—C8—C7	58.96 (13)	C6—C7—C8—Fe1	58.74 (13)
Fe1—C10—C6—C7	−60.07 (13)	C6—C7—C8—C9	−0.5 (2)
Fe1—C10—C11—C12	115.28 (17)	C8—C9—C10—Fe1	58.99 (12)
Fe1—C10—C11—C16	−67.2 (2)	C8—C9—C10—C6	−0.1 (2)
Fe1—C6—C7—C8	−59.44 (13)	C8—C9—C10—C11	−179.60 (16)
Fe1—C7—C8—C9	−59.24 (13)	C11—C12—C13—C14	−0.4 (3)
Fe1—C2—C1—C5	−59.75 (13)	C11—C10—C6—Fe1	−120.64 (17)
Fe1—C2—C3—C4	59.70 (13)	C11—C10—C6—C7	179.30 (16)
Fe1—C1—C5—C4	−59.11 (13)	C17—N1—C18—C19	0.5 (2)
Fe1—C5—C4—C3	−59.13 (13)	C17—N2—C20—C21	−151.80 (19)
Fe1—C4—C3—C2	−59.56 (13)	C17—N2—C19—C18	0.0 (2)
C12—C13—C14—N1	−177.24 (15)	C19—N2—C20—C21	33.4 (3)
C12—C13—C14—C15	0.8 (3)	C19—N2—C17—N1	0.3 (2)
C12—C11—C16—C15	1.0 (3)	C18—N1—C17—N2	−0.5 (2)
C13—C12—C11—C10	177.03 (16)	C21—C26—C25—C24	2.0 (3)
C13—C12—C11—C16	−0.5 (3)	C26—C21—C22—C23	0.3 (3)
C13—C14—N1—C17	−38.3 (3)	C26—C25—C24—N3	−178.53 (16)
C13—C14—N1—C18	140.25 (18)	C26—C25—C24—C23	0.0 (3)
C13—C14—C15—C16	−0.3 (3)	C25—C24—N3—O1	−162.01 (18)
C14—N1—C17—N2	178.30 (16)	C25—C24—N3—O2	19.5 (3)
C14—N1—C18—C19	−178.32 (16)	C25—C24—C23—C22	−1.8 (3)
C14—C15—C16—C11	−0.6 (3)	C24—C23—C22—C21	1.6 (3)
N1—C14—C15—C16	177.73 (16)	N3—C24—C23—C22	176.75 (17)
N2—C20—C21—C26	−86.3 (2)	C23—C24—N3—O1	19.4 (3)
N2—C20—C21—C22	94.7 (2)	C23—C24—N3—O2	−159.16 (19)
N2—C19—C18—N1	−0.3 (2)	C22—C21—C26—C25	−2.2 (3)
C20—N2—C17—N1	−175.29 (18)	C15—C14—N1—C17	143.62 (19)
C20—N2—C19—C18	175.42 (19)	C15—C14—N1—C18	−37.8 (3)
C20—C21—C26—C25	178.84 (17)	C2—C1—C5—Fe1	59.57 (13)
C20—C21—C22—C23	179.31 (17)	C2—C1—C5—C4	0.5 (2)
C9—C10—C6—Fe1	59.87 (12)	C1—C2—C3—Fe1	−59.25 (13)
C9—C10—C6—C7	−0.2 (2)	C1—C2—C3—C4	0.4 (2)
C9—C10—C11—C12	23.5 (3)	C1—C5—C4—Fe1	58.95 (13)
C9—C10—C11—C16	−159.04 (17)	C1—C5—C4—C3	−0.2 (2)
C10—C9—C8—Fe1	−58.58 (12)	C5—C4—C3—Fe1	59.40 (13)
C10—C9—C8—C7	0.4 (2)	C5—C4—C3—C2	−0.2 (2)
C10—C6—C7—Fe1	59.87 (12)	C3—C2—C1—Fe1	59.18 (13)
C10—C6—C7—C8	0.4 (2)	C3—C2—C1—C5	−0.6 (2)
C10—C11—C16—C15	−176.56 (17)		

### 3-(4-Ferrocenylphenyl)-1-(4-nitrobenzyl)-1H-imidazol-3-ium hexafluoridophosphate Hydrogen-bond geometry (Å, º)

**Table:**

*D*—H···*A*	*D*—H	H···*A*	*D*···*A*	*D*—H···*A*
C17—H17···F4^i^	0.95	2.29	3.208 (2)	163
C17—H17···F6^i^	0.95	2.34	3.115 (2)	138
C18—H18···F3	0.95	2.30	3.207 (2)	158
C15—H15···F3	0.95	2.53	3.216 (2)	129

Symmetry code: (i) *x*, −*y*+3/2, *z*−1/2.
